# Medical News and Miscellany

**Published:** 1891-02

**Authors:** 


### Medical News and Miscellany.

---

Dr. J. B. Rainey, of Gilmer, Texas, died Dec. 11.

---

You may know how hard anything is hit (a snake for instance) by the way it *squirms*. See?

---

Dr. F. E. Young, of Brownwood, has gone to New York to attend the Polyclinic; thence he will go to Berlin, in May or June, to attend the great clinics.

---

Libel Suit.—A Mr. Ready, of Bonham, has instituted suit against the Health Journal Publishing Company, at Dallas—Drs. J. R. Briggs and A. M. Elmore—for libel and damages, fixing the latter at $15,000.

**Dr. C. F. Paine**, Comanche, Chairman Section on Gynecology, Texas State Medical Association, requests us to say that he solicits papers for his Section, for the Waco meeting. Address him, or Dr. F. E. Young, Secretary, Brownwood.

---

**Dr. T. J. Wagley**, of Cleburne, Texas, has just returned from an extended tour through Europe. He attended the Tenth International Medical Congress at Berlin last August, and since that time has put in his time at the several great medical centers.

---

**Death of Dr. Terhune.**—The JOURNAL is pained to hear of the death of Dr. A. A. Terhune, sr., of Jefferson, Texas. He was one of the pioneer physicians of East Texas, and an old member of the State Medical Association. Particulars of his death are not learned at time of going to press.

---

**Dr. Geo. Cupples**, Chairman of the Committee on Medical Legislation T. S. M. A., has been very ill with pneumonia at his home in San Antonio, and at present, Feb. 19, he is barely convalescent. His numerous friends will join the JOURNAL in the hope that he will be spared many more years of usefulness to his fellow-men and to the medical profession.

---

**Injured Innocence.**—

"As vonce I valked by a dismal swamp
There sot an old cove in the dark and damp,
And at everybody as passed that road
A stick or a stone this old cove throwed;
And venever he flung his stick or his stone
He'd set up a song of "Let me alone."

Did any one see an 'old cove' at the Ft. Worth meeting throwing stones at anybody? And who *now* wants to be "let alone?"

---

**Quarantine Appointments.**—The following officers have been appointed by State Health Officer Swearingen:

Coast stations—A. N. Perkins, M. D., Sabine Pass; W. F. Blunt, M. D., Galveston; E. S. Weiseger, M. D., Velasco;

Thomas G. Duncan, M. D., Pass Cavallo; J. W. Irion, M. D., Aransas Pass; Arthus S. Wolff, M. D., Brazos Santiago.

Rio Grande inspecting stations—Fred J. Combe, M. D., Brownsville; Thomas J. Turpin, M. D., Laredo; W. M. Yandell, M. D., El Paso.

---

**The Marion-Sims Medical College.**—The new candidate for honors at the hands of the medical profession and for a share of the Texas patronage, has a full page advertisement in this issue. By reference to it the faculty will be seen to be composed of many of the ablest and best known medical men of the West—men who have become identified with their respective branches, and whose ability is universally recognized. The College starts off with great eclat, and opens the campaign under the most favorable auspices. Write to Dr. Y. D. Bond for a catalogue and mention the JOURNAL. If you have a student to send there, give him a letter to Dr. Love and to Dr. Bond, and the JOURNAL will guarantee a cordial reception and ample satisfaction.

---

**Asylum Appointments.**—Dr. Reeves, Superintendent of the (Austin) Lunatic Asylum has appointed Dr. B. M. Worsham, of Waxahachie, first, and Dr. T. O. Maxwell, of Travis county, second assistant physician at the asylum.

Dr. Dorset, late superintendent at the State Lunatic Asylum has returned to Bonham, his old home, and Dr. R. R. Walker, his first assistant, has returned to Paris, Texas.

Dr. B. F. Church, of Austin, has been appointed assistant physician to the State Lunatic Asylum at Terrell. Dr. Church is a graduate of the Baltimore Medical College, and a young man who came to Austin about two years ago. By his modest and gentlemanly deportment and studious habits, he made many friends and won the respect and kindly aid of the profession. The appointment tendered him unsolicited, is a high compliment, and worthily bestowed.

---

**Dangerous Proprietary Compounds.**—On account of the prevalent belief that the use of opium was rapidly increasing in Massachusetts, the Board of Health last year made an investigation to ascertain the extent of the evils arising therefrom. It procured and analyzed twenty-five of the most commonly used patent medicines, and among them all found only one which did not contain opium! On the list of those containing it was "Boschee's German Syrup of Tar," "Ayer's Cherry Pectoral," "Dr. Bull's Celebrated Cough Syrup," "Seth Arnold's Cough Killer," "Perry Davis' Pain Killer," "Dr. Hooker's Cough and Croup Syrup," "Mrs. Winslow's Soothing Syrup," "Mother Bailey's Quieting Syrup," and "Dr. Wistar's Balsam of Wild Cherry." The Board of Health in summarizing the results of its investigation uses these words: "The sale of soothing syrups and all medicines designed for the use of childen which contain opium in its preparation, should be prohibited."

---

Bright and Sparkling is the *Cosmopolitan;* moreover, it is the cheapest illustrated monthly (and one of the very best) published anywhere. The subscription price is only $2.40, but clubbed with DANIEL'S TEXAS MEDICAL JOURNAL it will cost you only $1.35. How is that? Send $3.35 to this office, and get both bright and sparkling publications for a year.

The *Medical Mirror*, St. Louis, "b. & s." also, $2 a year. DANIEL'S TEXAS MEDICAL JOURNAL and the *Mirror* one year for $3. How is that?

The *New Orleans Medical and Surgical Journal* $3. DANIEL'S TEXAS MEDICAL JOURNAL and the *New Orleans Medical and Surgical Journal*, $4.

DANIEL'S TEXAS MEDICAL JOURNAL and the *Brooklyn Medical Journal*, both for $3.50.

DANIEL'S TEXAS MEDICAL JOURNAL and *Lamphear's Kansas City Medical Index*, both for $3.50.

The above is to new subscribers only. Money sent by express must be prepaid. No local checks taken; we have to discount them.

---

The American Electro-Therapeutic Association was organized on the 22nd of January, 1891, at the Academy of Medicine, No. 17 West 43rd street, New York, by the adoption of a constitution and by-laws, and the election of the following officers: President, G. Betton Massey, M. D., Philadelphia; Vice-Presidents, William James Norton, M. D., and Augustus H. Geolet, M. D., New York; Secretary, William H. Walling, M. D., Philadelphia; Treasurer, Geo. H. Rohe, M. D., Baltimore.

*Executive Council.*—Horatio R. Bigelow, M. D., Philadelphia- Franklin H. Martin, M. D., Chicago; W. F. Hutchinson, M. D., Providence, R. I.; Frederic Peterson, M. D., New York, and Chauncey D. Palmer, M. D., Cincinnati, Ohio.

The object of the Association, as stated in Art. 2 of the Constitution is "the cultivation and promotion of knowledge in whatever relates to the application of electricity in medicine and surgery."

The Association starts with a strong and vigorous membership, and has every prospect of a most useful and successful career.

The next meeting will be held in Philadelphia, in September of this year. WM. H. WALLING, M. D., Secretary,
2005 Arch St., Philadelphia, Pa.

---

**To Delinquent Members Texas State Medical Association:**—Notice is hereby given that the Secretary has on hand one hundred and fifty-six copies of the last Transactions, wrapped and addressed, but held because of non-payment of dues for 1890-1. Under resolution adopted at Fort Worth he was instructed to send the Transactions only to those who paid their dues—and of the five hundred there are one hundred and fifty-six whose name are not on the list furnished him by the Treasurer. Each one who has not received a copy, will know why, and those who have not paid know they have not paid, without giving names. The Secretary is anxious to get rid of this lot of Transactions, and again calls on these members to pay up. Remit to Dr. Larendon, Treasurer, Houston, and on receipts of a postal card from him the Secretary will send you a copy of the Transactions.

The rule adopted at last meeting of giving badges and admittance to those only who pay dues, will be still more rigidly enforced at Waco than it was at Fort Worth. A word to the wise is sufficient. The Treasurer reports this year's collections ahead of any other year, and it is clearly due to the adoption of the above policy.

**Legislative Notes.**—A billto compel vaccinnation has been reported favorably by the House Committee on Public Health.

**Dr. Pope's** bill to create a College of Physicians and Surgeons in Texas,—a synopsis of which was published in our October issue,—has been returned to the House by the Committee on Public Health with the recommendation that it do not pass.

**A Bill** amending the pharmacy law, so as to extend its operations to the cross-roads and smaller towns—thus requiring examinations of the drug cub who sells drugs along with dry goods and groceries—was also returned with a black eye; as was also an amendment proposed to the law regulating the practice of dentistry—tightening its grip.

**Doctors as Legislators.**—In the House of the 22nd Texas Legislature, now in session, there are some four or five doctors—all "regular." Dr. Selman, of Tyler, is chairman of the Committee on Public Health and Vital Statistics, and he has as colleagues Drs. L. Lloyd, of Jacksonvile; A. C. Oliver, of Cass Co.; Geo. F. Perry, of Hamilton Co., and J. D. McGregor, of Austin county.

**A State Veterinarian,**—One of the most important measures proposed at the present session of the Legislature was introduced on the 12th inst. It provides for the appointment of a State Veterinarian, with a salary of $1500, and expenses while on duty. His special functions, as defined by section 3, is "to protect the health of the horse, ass and mule and other stock from contagious diseases," and to that end he has authority to condemn to death any animal infected with contagious diseases, payment for the same being secured to the owner by appraisement by three disinterested stockmen or farmers. The object sought is the prevention or suppression of contagious diseases amongst stock. It really seems that more importance is attached to the health of the cattle than to that of the citizens and tax payers.

**The** "Bill to Regulate the Practice of medicine and to create a Board of Medical Examiners in Texas," prepared by the Committee on Medical Legislation of the Texas State Med. Association, was introduced into the Texas Senate by Sen. Pope on Feb. 14th, and was referred to the Committee on Public Health. It was reported upon favorably by the committee, and recommended for passage with an amendment striking out section 10 which relates to revoking licenses for unprofessional conduct. It will go the House shortly and will, in all probability, become a law.

It seems very difficult to make the legislators comprehend that there is any danger in the indiscriminate practice of medicine, and that sentiment "this is a free country" seems to extend to the privilege of practicing medicine. Many say that a sick man has a right to send for whom he pleases. No doubt of that, but should not every man who essays to engage in the practice of medicine, give to a constituted authority evidence that he understands, at least, the general principles—and has been edudated to the calling? evidence of fitness, no matter how or where acquired? We think so. The Homeopaths claim that no danger attends the administration of their remedies.(?) As the "administration of their remedies" is tantamount to doing nothing—the danger is one of *omission*—the failure to do somthing and the proper thing. But—how many of them confine their practice to "the administration of homeopathic remedies"? Not one out of fifty; and their ignorance of the therapeutics of the "regulars" or "old school," as they call us, which they essay to practice, constitutes a grave danger. No, there is no danger in homeopathic "remedies."(?)

